# Data-Driven Selection of Parametric and Travelling Fire Models for Performance-based Fire Design

**DOI:** 10.1007/s10694-026-02017-8

**Published:** 2026-10-14

**Authors:** Qingfeng Xu, Mohammad Javad Moradi, Hamzeh Hajiloo, Charlie Hopkin, Jun Heng Low, Lee Cunningham, Yong Wang

**Affiliations:** 1 Department of Civil Engineering and Management, University of Manchester, Manchester, United Kingdom; 2 Ashton Fire, Manchester, United Kingdom; 3 Department of Civil and Environmental Engineering, Carleton University, Ottawa, Canada

**Keywords:** Performance-based design, Compartment fires, Full-scale fire experiments, Logistic regression, Travelling fire

## Abstract

Selecting appropriate design fire models remains a key unresolved challenge in performance-based structural fire engineering, where the applicability of the Eurocode EN 1991-1-2 parametric fire curve and travelling fire (TF) models is not clearly defined. This study proposes a quantitative threshold for selecting between the EN and TF models based on full-scale experimental results. A total of 222 available full-scale natural fire experiments are summarised and used as benchmark data. For each experiment, average compartment fire temperature–time curves are predicted using the EN and TF models, respectively, and their predictive accuracy is quantitatively assessed to determine the best-performing model for each fire test. A logistic regression (LR) model is then derived from the available dataset to predict the best-performing model for a given set of fire inputs. As the required TF model inputs, namely the peak fire temperature, heat release rate per unit area, and fire spread rate, were not reported in the experimental data, a sensitivity study is conducted to evaluate their influence on the derived LR model. The results show that the LR model is most sensitive to the assumed fire spread rate. Therefore, three applicability segments are introduced into the LR model to cover a wider range of fire scenarios. The proposed LR model achieves an average training accuracy of 0.736 and an ROC–AUC of 0.808. A probabilistic risk assessment case study using Monte Carlo simulation demonstrates that the proposed LR model reduces both over- and under-design compared with EN-only or TF-only methods, providing a more balanced and reliable framework for current performance-based structural fire design.

## Introduction

With the development of new structural systems, prescriptive regulations cannot cover all of them. Therefore, performance-based design offers a more flexible approach to structural engineering, ensuring sufficient safety while avoiding unnecessary resource expenditure [1]. As an example, the ISO-834 standard fire curve [2] is commonly used to determine the fire resistance period of structures. However, it does not represent real fire conditions, as it neglects the influence of ventilation, fire load, compartment geometry, and the decay phase [3, 4]. These factors can significantly affect fire severity and lead to substantially different temperature–time curves [5, 6]. Therefore, identifying potential fire scenarios within a building is essential when determining an appropriate fire performance time.

To address the limitations of the ISO standard fire curve, the European project Natural Fire Safety Concept (NFSC) was undertaken to replace it with more realistic scenarios, namely natural fires [7]. A series of large-scale fire experiments was conducted by the UK’s Building Research Establishment (BRE) to understand and validate the behaviour of natural fires in real-world compartments. The findings from these tests further informed the development of the Eurocodes (e.g., EC1 and EC3) and demonstrated the importance of considering structural system behaviour in fire. They also highlighted the influence of fire load, ventilation conditions, and compartment geometry on compartment fire development. Subsequent research, such as the travelling fire (TF) methodology developed by Rein et al. [8], proposed that in large compartments, fires may burn locally and spread progressively across the floor over time. This concept was based on observed fire dynamics from real incidents and supported by several experimental programmes over the past two decades [9–12].

The most accurate way to determine fire behaviour is through full-scale fire experiments, as they capture realistic physical fire dynamics and their interaction with structural systems [13]. For example, BRE conducted six large-scale fire tests on steel-framed buildings at Cardington between 1994 and 1997, which became the foundation for modern structural fire engineering codes and performance-based design methods still in use today [14]. The Cardington tests demonstrated that buildings possess inherent fire performance through structural continuity and redundancy, which isolated member testing cannot reveal. Although full-scale fire experiments have provided invaluable insights into fire science, they cannot be readily replicated because they are disproportionately expensive and practically impossible to conduct across all possible fire scenarios and compartment configurations. This limitation has motivated the development of various numerical and empirical fire models that aim to reproduce the thermal behaviour of real fires with different levels of approximation [15].

Although the EN 1991-1-2 parametric fire curve [16] and the TF model are both natural fire models used to characterise thermal exposure in compartment fire analyses, the accuracy of their applicability limits is not clear, and guidance on selecting the most appropriate model for a given compartment is lacking. The EN fire model is assumed to be valid for compartments with floor areas up to 500 [m$^2$]. However, previous full-scale fire experiments have shown that, as compartment size increases, fire behaviour tends to transition towards a TF rather than a uniform post-flashover fire [9, 17, 18], since flashover becomes less likely under such conditions. Although efforts have been made to validate the EN fire curve using full-scale fire tests [19], these studies were limited to compartments of up to 100 [m$^2$] and did not explicitly consider variations of other key fire parameters, such as fire load density, ventilation conditions, and thermal inertia. As a result, a practical and systematic tool is needed to distinguish between scenarios where the EN parametric fire curve is appropriate and those where the TF model should be used in fire design.

With the development of data-driven methods, statistical and machine learning techniques are increasingly used to identify implicit relationships between multiple parameters. For example, experimental datasets can be used to distinguish between EN and TF using classification models such as logistic regression (LR), which provides a robust and interpretable framework for binary classification [20]. Binary LR is a statistical classification method that estimates the probability that an outcome belongs to one of two classes, given a set of input variables. One of the most common applications of LR in fire engineering is the prediction of fire occurrence, particularly for wildfires using image or spatial data [21, 22]. With sufficient statistical data, LR has also been shown to be effective in identifying key factors that contribute most significantly to target outcomes, such as injury or structural failure [23, 24]. These studies demonstrate the capability of LR for both prediction and classification tasks. Building on these strengths and informed by prior full-scale fire experiments, this study aims to develop a LR–based framework to identify an appropriate threshold for distinguishing between the EN parametric fire and TF models, thereby addressing a key challenge in performance-based structural fire engineering and fire risk assessment.

In this study, results from 222 available full-scale natural fire experiments are summarised and used as benchmark data. Based on the reported test setups, both the EN parametric fire model and the TF model are used to predict the corresponding temperature–time curves, and their prediction accuracy is evaluated to find the best-performing model. The results are then analysed using a LR model to identify correlations between the experimental parameters and the best-performing fire model for specific fire scenarios. Once trained, the model outputs a threshold for separating the EN parametric fire curves and the TF model.

The structure of this paper is as follows: Sect. 2 introduces the methodology and theoretical background of this paper. Sect. 3 presents the collected dataset and the preprocessing steps for training the LR model, and Sect. 4 evaluates the model performance. Sect. 5 demonstrates how the LR model can be incorporated into structural fire design to support decision-making. Finally, Sect. 6 summarises the key findings and recommendations of this study.

## Methodology and Theoretical Background

This section introduces the theoretical background of the methods used in fire risk calculations, together with the proposed LR model: (a) the Eurocode 1991-1-2 parametric fire curves [16] and the travelling fire model [17, 25, 26]; (b) lumped-mass steel temperature calculations in accordance with Eurocode 1993-1-2 [27]; (c) the sensitivity study to identify key parameters in natural fire curve; (d) the LR model; and (e) the Monte Carlo simulation.

### Data-Driven Logistic Regression Model Based on Full-Scale Tests

**Fig. 1:**
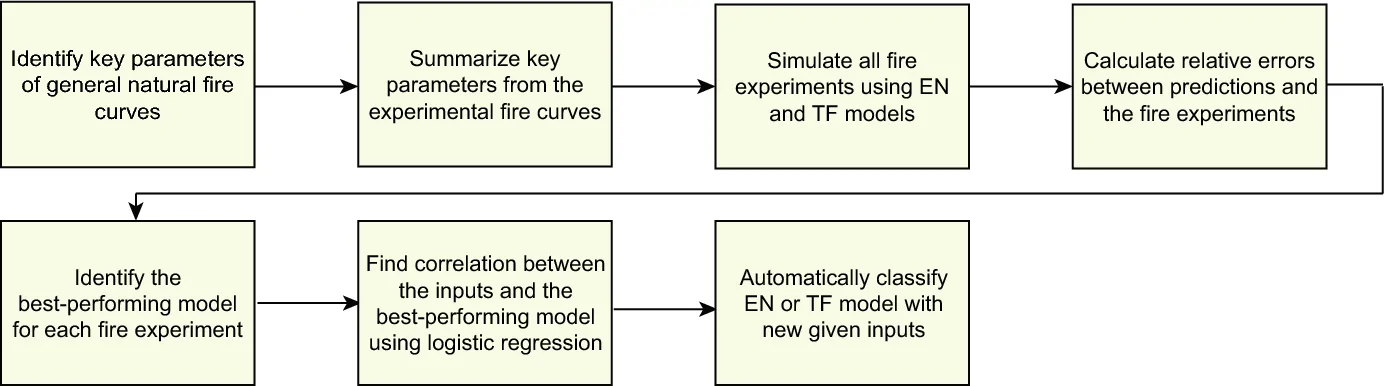
Flowchart of the proposed data-driven LR model framework

The procedure for deriving the data-driven LR model used to classify the EN and TF models is illustrated in Fig. 1. Because a direct comparison of fire curves is difficult, a criterion is first needed to determine which model gives better predictions in natural fire tests. As shown in the first step, this is achieved by conducting a sensitivity study to identify the key parameters of a general natural fire curve, based on their influence on the peak steel temperature. These parameters are then used to assess the accuracy of fire model predictions for a given fire test.

In the second step, a database of full-scale fire experiments is created by collecting as many such experiments as possible. For each experiment, the experimental setup, measured average fire temperature-time curves, and the key parameters identified in the sensitivity analysis are summarised for later analyses.

In the third step, each fire test is simulated using both the EN fire curve and the TF model. The corresponding predicted temperature–time curves are then obtained for comparison.

In the fourth step, the relative errors between the predicted and experimental values of the identified key parameters are calculated to quantitatively evaluate the predictive accuracy of the EN and TF models and identify the best-performing model for each test.

Finally, a LR model is used to identify the hidden relationships between the experimental setups (inputs) and the best-performing fire model (outputs). Once trained, this model can be used to automatically predict the most suitable fire model for a given set of input parameters.

### Fire Curves and Lumped Mass Steel Temperature Calculation

#### BS EN 1991-1-2 Parametric Fire Curves

The EN parametric fire model provides a simplified method for predicting fire temperature–time curves that account for physical factors influencing fire development inside a compartment, such as the compartment geometry, the fire load, the size of wall openings, and the thermal properties of the construction materials forming the compartment boundaries [28, 29]. The governing equation is shown below:

1 $$\begin{aligned} T_g = 20+1325\left( 1-0.324e^{-0.2t^*}-0.204e^{-1.7t^*}-0.472e^{-19t^*}\right) \end{aligned}$$

where $T_g$ [°C] is the gas temperature in the fire compartment, $t^*=t\cdot \Gamma$ [h] is the characterised time corresponding to the time *t* [h], and *Γ * is a non-dimensional time factor, which can be calculated as follows:

2 $$\begin{aligned} \Gamma = (O/b)^2/(0.04/1160)^2 \end{aligned}$$

*O* [m$^{0.5}$] is the opening factor, and *b* [J/m$^2$·s$^{1/2}$·K] is the thermal inertia. The peak gas temperature ($T_{max}$) occurs when $t^* = t^*_{max}$, with:

3 $$\begin{aligned} {\left\{ \begin{array}{ll} t^*_{max} = t_{max}\cdot \Gamma \\ t_{max}=\max \left[ \left( 0.2\cdot 10^{-3}\cdot q_{t,d}/O\right) ,\ t_{lim}\right] \end{array}\right. } \end{aligned}$$

Here, $q_{t,d}$ [MJ/m$^2$] is the design value of the fire load density referred to the total surface area of the compartment. The parameter $t_{lim}$ [h] is the fire growth time, with $t_{lim} = 15$, 20, and 25 [min] for fast, medium, and slow fire growth rates, respectively. After the peak temperature is reached, the cooling phase is defined by:

4 $$\begin{aligned} T_g = {\left\{ \begin{array}{ll} T_{\max } - 625 \left( t^* - t_{\max }^* \cdot x\right) , & \text {for } t_{\max }^* \le 0.5, \\ T_{\max } - 250 \left( 3 - t_{\max }^*\right) \left( t^* - t_{\max }^* \cdot x\right) , & \text {for } 0.5< t_{\max }^* < 2, \\ T_{\max } - 250 \left( t^* - t_{\max }^* \cdot x\right) , & \text {for } t_{\max }^* \ge 2. \end{array}\right. } \end{aligned}$$

with *x* is calculated as follows:

5 $$\begin{aligned} x = {\left\{ \begin{array}{ll} 1.0 & \text {for } t_{\max } > t_{lim}, \\ t_{lim}\cdot \Gamma /t_{\max }^* & \text {for } t_{\max } = t_{lim} \end{array}\right. } \end{aligned}$$

The EN fire model is based on the assumption that the temperature within the compartment is spatially uniform. As a result, its applicability is generally limited to post-flashover fires. The following restrictions apply: (a) the design fire load density $q_{t,d}$ must be between 50 and 1000 [MJ/m$^2$], and the fuel must be limited to cellulosic-type fuels [16, 30]; (b) the maximum floor area is 500 [m$^2$]; (c) the compartment thermal inertia *b* ranges between 100 and 2200 [J/m$^2$·s$^{0.5}$·K]; (d) the opening factor must be within the range of 0.02 to 0.2 [m$^{0.5}$]; and (e) the compartment height must not exceed 4 [m].

#### Travelling Fire Curves

**Fig. 2:**
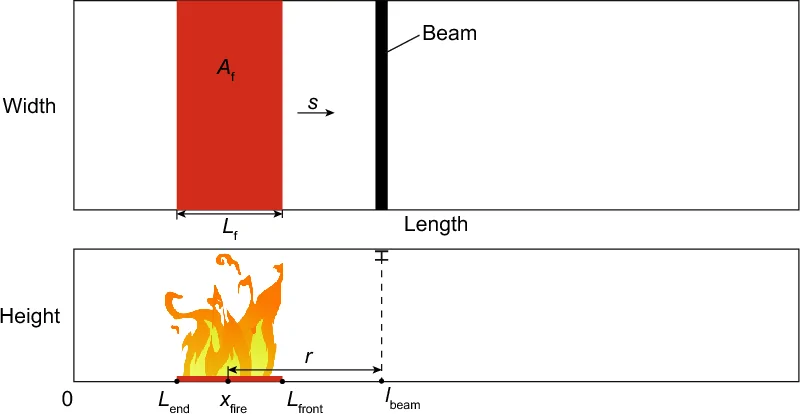
Travelling fire model explanation

The travelling fire model assumes compartment fires are spatially non-uniform and developing in space and time. The original travelling fire model was proposed by Alpert [31] and further improved by Stern-Gottfried et al. [17] and Rackauskaitė et al. [32]. In this study, a further development of the model proposed by Hopkin et al. [26] and Fu et al. [33] is used. In this approach, the fire is modelled as a localized burning region that moves across the floor area as combustible fuel is progressively consumed, see Fig. 2. The governing equations of the model are summarised below.

The travelling fire is assumed to be fuel-controlled at all times. Consequently, the local burning time $t_b$ [s] is determined by the floor fire load density $q_f$ [MJ/m$^2$] and the heat release rate per unit area $\dot{Q}''$ [MW/m$^2$]:

6 $$\begin{aligned} t_b = \frac{q_f}{\dot{Q}''} \end{aligned}$$

Realistic fire spread rates for different fuel types are reported by Table 1 of Rackauskaitė et al. [32]. Given a constant fire spread rate *s* [m/s], the position of the leading edge of the fire at time *t* [s] is defined as:

7 $$\begin{aligned} L_{\textrm{front}} = \min [s \cdot t, L] \end{aligned}$$

where *L* [m] is the length of the compartment. And the ending edge of the fire can be calculated by:

8 $$\begin{aligned} L_{\textrm{end}} = \min [s \cdot (t - t_{\textrm{lim, TF}}), L] \end{aligned}$$

where the limiting burning duration $t_{lim, TF}$ is given by:

9 $$\begin{aligned} t_{\textrm{lim, TF}} = \min \left[ t_b, \frac{L}{s}\right] \end{aligned}$$

The instantaneous length of the travelling fire is therefore:

10 $$\begin{aligned} L_f = L_{\textrm{front}} - L_{\textrm{end}} \end{aligned}$$

The centre of the fire is assumed to be the midpoint between the leading and ending edges:

11 $$\begin{aligned} x_{\textrm{fire}} = \frac{L_{\textrm{front}} + L_{\textrm{end}}}{2} \end{aligned}$$

The burning area $A_f$ [m$^2$] is assumed to occur on the full compartment width *w* [m]:

12 $$\begin{aligned} A_{f} = L_{f}\cdot w \end{aligned}$$

The total heat release rate $\dot{Q}$ [MW] is then calculated as:

13 $$\begin{aligned} \dot{Q} = A_f \cdot \dot{Q}'' \end{aligned}$$

The gas temperatures at the interested point (e.g., a beam located in the ceiling) are divided into two regions: (a) the near-field, and (b) the far-field. The near-field corresponds to regions where flames directly impinge on the ceiling, and the gas temperature is assumed to reach the peak flame temperature. The far-field is characterized by a hot smoke layer, with temperatures decreasing with increasing distance from the fire source.

For the far-field, the maximum gas temperature $T_{\max }$ [°C] is calculated using Eq. 14:

14 $$\begin{aligned} T_{\max } - T_{\infty } = 5.38 \frac{\left( \dot{Q} / r \right) ^{2/3}}{H}, \hspace{20pt}\text {for} \frac{r}{H} \ge 0.18 \end{aligned}$$

where $T_{\infty }$ [°C] is the ambient room temperature, *r* [m] is the radial distance from the fire source, and *H* [m] is the compartment height.

For the near-field, the maximum gas temperature $T_{\max }$ [°C] is calculated using Eq. 15:

15 $$\begin{aligned} T_{\max } - T_{\infty } = 16.9 \frac{\left( \dot{Q} \right) ^{2/3}}{H^{5/3}}, \hspace{20pt}\text {for} \frac{r}{H} < 0.18 \end{aligned}$$

Finally, the gas temperature $T_{g}$ is limited by a maximum flame temperature:

16 $$\begin{aligned} T_{\textrm{g}} = \min [T_{\max }, T_{\max ,\textrm{lim}}] \end{aligned}$$

where $T_{\max ,\textrm{lim}}$ typically ranges between 800 and 1200 [°C].

#### Lumped Mass Steel Temperature Calculation

For fire-protected steel members, the temperature of the fire-exposed steel is calculated following the Eurocode EN 1993-1-2 [27]. Assuming a uniform temperature distribution across the steel cross-section, the temperature increase $\Delta T_{s,t}$ [$^\circ$C] over a time interval *Δ t* [s] is given by Eq. 17.

17 $$\begin{aligned} \Delta T_{s,t} = \frac{\lambda _p A_p / V \, (T_{g,t} - T_{s,t})}{d_p c_s \rho _s \, (1 + \phi /3)} \Delta t - \left( e^{\phi /10} - 1 \right) \Delta T_{g,t} \end{aligned}$$

with the dimensionless parameter *ϕ *:

18 $$\begin{aligned} \phi = \frac{c_p \rho _p}{c_s \rho _s} d_p A_p / V \end{aligned}$$

where $A_p / V$ [m$^{-1}$] is the section factor of the steel member, while $c_s$ [J/(kg·K)] and $\rho _s$ [kg/m$^3$] are the specific heat and density of steel, respectively, as specified in Section 3 of EN 1993-1-2 [27]. $c_p$ [J/(kg·K)] and $\rho _p$ [kg/m$^3$] are the specific heat and density of the fire protection material. $d_p$ [m] is the thickness of the fire protection layer, $\lambda _p$ [W/(m·K)] is its thermal conductivity, and $T_{s,t}$ [°C] and $T_{g,t}$ [°C] are the temperatures of the steel and the gas at time *t*, respectively. $\Delta T_{g,t}$ [K] is the gas temperature increase during the interval *Δ t* [s].

### Identify Key Parameters of Natural Fire Curves

As mentioned in Sect. 2.1, given the results of a natural fire test, it is still difficult to quantitatively assess the accuracy of different fire models. For example, a fire model may provide a good prediction of peak fire temperature, whereas it may differ in predicting total fire duration or time to flashover [34]. It is therefore important to identify the key parameters that characterise a natural fire, so that the relative errors of different model predictions can be quantitatively compared.

In this paper, a general natural fire curve is generated using polynomial functions in Python, so that the key characteristics of the curve (e.g. flashover, peak temperature, and cooling phase) can be readily adjusted to represent a wide range of fire scenarios [34].

The general fire curve is constructed as a piecewise, shape-controlled function passing through four control points: the ambient temperature at ignition (0, 20), the temperature at flashover $(t_1, T_1)$, the peak temperature at the end of the development phase $(t_2, T_2)$, and the residual temperature at the end of the cooling phase $(t_3, T_3)$. Each phase is represented by a power-law expression anchored at one control point, with the exponents $n_1$, $n_2$ and $n_3$ governing the curvature of the growth, fully developed, and cooling phases independently of the characteristic temperatures and times:

19 $$\begin{aligned} T(t) = {\left\{ \begin{array}{ll} a_1\,t^{\,n_1} + 20, & 0 \le t \le t_1 \\ T_2 - a_2\,(t-t_2)^{n_2}, & t_1< t \le t_2 \\ a_3\,(t-t_3)^{n_3} + T_3, & t_2 < t \le t_3 \\ T_3, & t > t_3 \end{array}\right. } \end{aligned}$$

where

20 $$\begin{aligned} a_1 = \frac{T_1-20}{t_1^{\,n_1}}, \qquad a_2 = \frac{T_2-T_1}{(t_2-t_1)^{n_2}}, \qquad a_3 = \frac{T_2-T_3}{(t_3-t_2)^{n_3}}. \end{aligned}$$

The growth, development and cooling exponents $n_1$, $n_2$ and $n_3$ are free parameters, allowing the fire growth rate, flashover sharpness and cooling rate to be varied independently. This formulation was adopted because it provides the flexibility required to evaluate the sensitivity of peak steel temperature to each stage of the fire separately. The generated fire curve is intended as a general representation of a natural fire and is used only for the sensitivity study. The proposed generated fire curve was benchmarked against the DIN EN 1991-1-2/NA and EN 1991-1-2 Annex A parametric fire curves for several representative compartment scenarios. The predicted peak steel temperatures differed by only 1–4%, indicating that the proposed representation provides comparable results for the purpose of the sensitivity analysis.

Based on the generated fire curve from Eq. 19, the corresponding steel temperature is calculated using Eq. 17. The influence of each fire-curve parameter is quantified by varying one parameter at a time and calculating the resulting percentage change in peak steel temperature.

**Fig. 3:**
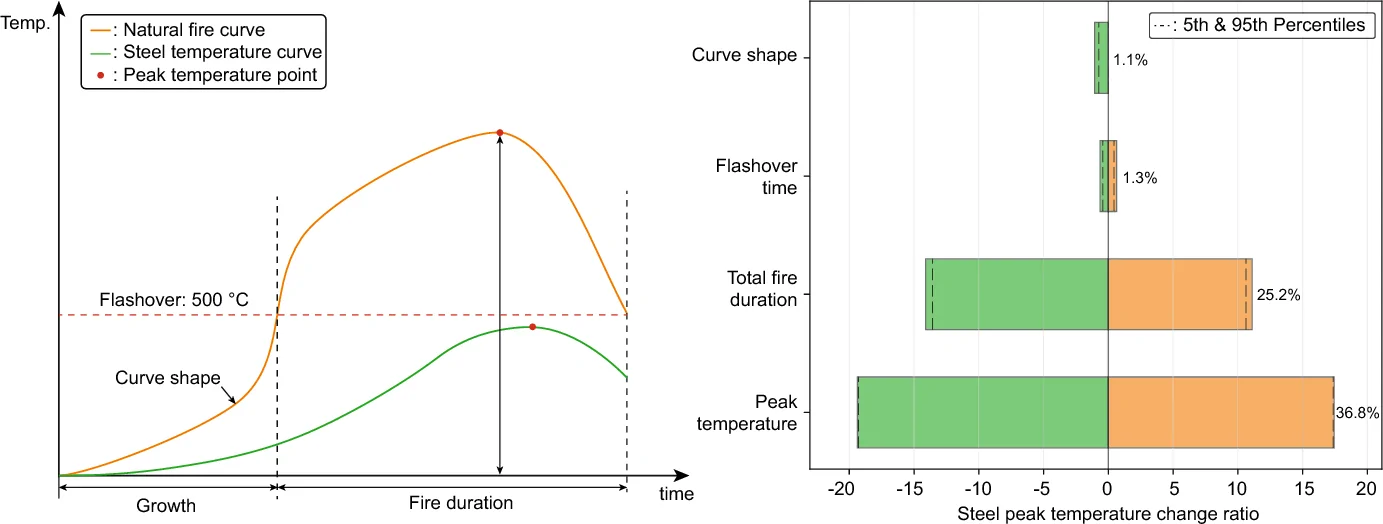
Parameters of natural fire curves and sensitivity analysis results

During a fire, structural failures most often occur during the post-flashover phase, and the peak steel temperature typically occurs shortly after the peak fire temperature. In addition, the fire growth phase is significantly different between the EN fire curve and the TF model. Therefore, four key features are selected for the sensitivity analysis as shown in Fig. 3 on the left and Table 1: (a) the peak fire temperature, (b) the duration for which the fire temperature remains above the flashover temperature (taken as 500 [°C] [35, 36]), (c) the time to reach flashover, and (d) the shape of the fire curve during the growth phase. Each parameter represents a single physical quantity – temperature, time, or curve shape, so that their individual influence on the structural response can be decoupled. Parameters (a) and (b) are supported by a prior sensitivity analysis [34], which identified peak fire temperature and fire duration as the dominant parameters governing peak steel temperature. Parameter (c) has comparatively little effect on the peak steel temperature itself but remains relevant to time-dependent analyses, such as determining the structural failure time. Parameter (d) is included primarily to confirm that the growth-phase shape does not contribute more than parameters (a) and (b) to changes in peak steel temperature, and to capture the difference in growth-phase behaviour between the EN fire curve and the TF model. For the material properties of the fire-protected steel members, the values are taken from a 90-minute fire-resistance design (according to ISO 834).

**Table 1:** Parameters for sensitivity analysis

Parameters	Referenced value	Range of data variation
Flashover time [min]	10	[5, 15]
Curve shape [-]	Linear	[Linear, quadratic]
Peak fire temperature [$^\circ$C]	1000	[750, 1250]
Total fire duration [min]	100	[80, 120]
Steel section factor ($A_p / V$) [m$^{-1}$]	116.18	Deterministic
Fire protection density ($\rho _s$) [kg/m$^3$]	350	Deterministic
Fire protection thermal conductivity ($\lambda _p$) [W/(m·K)]	0.03	Deterministic
Fire protection specific heat ($c_p$) [J/(kg·K)]	1000	Deterministic
Fire protection thickness ($d_p$) [m]	0.0043	Deterministic

For each parameter in the sensitivity analysis, a grid of test cases is generated in which the target parameter varies by around ± 25% across three levels (minimum, nominal, and maximum), as indicated in Table 1. Importantly, the sensitivity of each parameter is not evaluated around a single reference case: the variation is applied across all possible combinations of the other parameters within their uncertainty ranges, and the full analysis is further repeated for reference curves with different flashover temperatures [350–650 (°C)] and different times to reach the peak temperature (70–90% of the total fire duration). This yields approximately 160 simulation scenarios per parameter and captures the maximum sensitivity of the selected features across a family of fire scenarios, rather than being dependent on a single reference fire curve. The variation ratio of the peak steel temperature relative to the referenced fire curve is presented in Fig. 3 on the right. The steel section factor and fire protection properties (Table 1) are held deterministic, as they are fixed by the 90-minute fire-resistance design and define the structural configuration under assessment rather than uncertain fire-scenario inputs.

The results of the sensitivity analysis indicate that the peak fire temperature has the strongest influence on the peak steel temperature, accounting for 36.8% of the change across all scenarios, whereas the total fire duration accounts for 25.2%. These values, $W_T = 0.368$ and $W_d = 0.252$, are the weights of these two parameters. In contrast, the fire growth phase (i.e., before the flashover) contributes less than 1.5% changes and can therefore be considered negligible. Accordingly, when comparing model predictions with experimental fire temperature–time curves from the structural fire engineering perspective, the most important factor is to match the maximum fire temperature, followed by the total fire duration.

### Logistic Regression Model

LR is a statistical classification method commonly used to model the probability of a binary outcome as a function of explanatory variables. In this study, the fire model inputs are used to relate the likelihood of the best-performing fire model.

The LR model assumes that the combined effect of the input variables $x_1, x_2,..., x_n$ can be represented as a linear predictor *z*, expressed as a weighted sum of the inputs and a constant intercept term $\beta _0$, as shown in Eq. 21:

21 $$\begin{aligned} z = \beta _0 + \beta _1 x_1 + \beta _2 x_2 + \cdots + \beta _n x_n \end{aligned}$$

The coefficients $\beta _1, \beta _2,... \beta _n$ quantify the relative influence of each parameter on the model output and are determined through calibration using the available dataset. The linear predictor *z* is subsequently transformed into a probability using the logistic (sigmoid) function given in Eq. 22:

22 $$\begin{aligned} p = \frac{1}{1 + e^{-z}} \end{aligned}$$

This transformation ensures that the predicted probability is bounded between 0 and 1, making it suitable for representing the likelihood of occurrence of a binary event. In this study, the probability *p* is interpreted as the likelihood of the TF fire model under a given fire scenario. If *p ≥ 0.5*, then it predicts the TF fire model. Otherwise, it predicts the EN model.

### Monte Carlo Simulation and Failure Prediction

A large number of fire scenarios can be generated by randomly sampling from input parameter distributions using Monte Carlo simulations, and a deterministic analysis is performed for each generated scenario to evaluate the corresponding steel temperature. When all simulations (*N* in total) have been completed, the probability of failure, $P_f$, is estimated as the proportion of simulations in which the steel temperature exceeds a specified threshold. The failure probability is calculated as:

23 $$\begin{aligned} P_f = \frac{N_f}{N} \end{aligned}$$

where $N_f$ is the number of simulations resulting in failure and *N* is the total number of simulations. As the number of simulations increases, the estimated failure probability converges to the true failure probability, provided that the input distributions adequately represent the possible fire scenarios.

## Data Collection and Processing

### Data Description

**Fig. 4:**
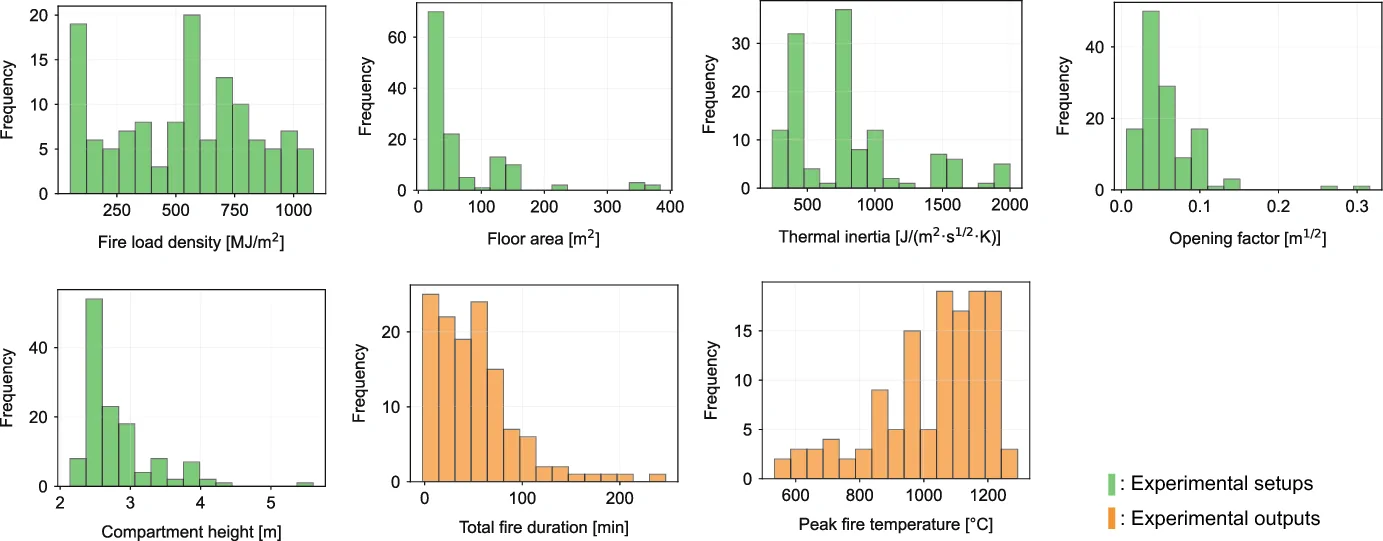
Histograms of the selected database from full-scale fire experiments

To obtain a sufficiently large dataset for training the LR model, this study has collected data from 222 full-scale compartment fire tests, some of which are summarised by Moradi et al. [37]. For each test, key experimental setups are recorded, including compartment geometry (length, width, and height), material properties of the lining (density, thermal conductivity, and specific heat capacity), ventilation factors, and fire-load density. The measured average gas temperature–time curves within the compartments are also extracted and post-processed to obtain essential fire characteristics. Only full-scale compartment fire experiments were included in the original dataset, with “large-scale” defined as compartments having a floor area greater than 10 [m$^2$]. The fuel types were restricted to materials that are progressively consumed during combustion, excluding continuously supplied fuels such as burning gases or burners. In addition, the dataset considered only naturally ventilated compartments and did not account for the influence of mechanical ventilation. The distributions of the aforementioned parameters are shown in Fig. 4.

The collected database covers a wide range of fire scenarios that may occur in compartments. The floor fire load density $q_{f}$ ranges from 50 to 1085 [MJ/m$^2$], covering nearly 99.94% of the fire load density range for office buildings recommended by Eurocode 1991-1-2 [16], which suggests a Gumbel distribution with a mean value of 420 [MJ/m$^2$] and a coefficient of variation (COV) of 0.3. The floor area of the experimental tests extends up to 383 [m$^2$]; however, most tests focus on compartments with floor areas smaller than 100 [m$^2$] due to the high costs. These compartments represent a substantial portion of real-world situations, such as small apartments, studio units, hotel rooms, and individual rooms within larger residential or office buildings [30].

The dataset includes a wide range of compartment lining materials, from gypsum boards to concrete, as reflected by the characterised thermal inertia values. The opening factor is calculated based on the compartment openings (e.g. open doors or glazing breakage in real fires) and ranges from 0.0005 to 0.36 [m$^{1/2}$]. This range covers ventilation conditions from poorly ventilated to better ventilated compartments, as tested by Kirby [9], to open-ventilation conditions, as investigated by Alam et al. [38]. The compartment height is generally consistent with typical compartments, ranging from 2.8 to 5.5 [m].

Since both EN and the TF model are not intended to reproduce the exact local gas temperature history measured at a specific location within a compartment, but rather to represent the overall thermal severity of a compartment fire for structural fire engineering purposes, several assumptions are made here to enable comparison with the fire tests: (a) the compartment floor area is assumed to be square; (b) the measurement location for the TF model is assumed to be at the centre of the compartment; and (c) averaged compartment temperature–time curves are used when multiple measurement locations were reported. These assumptions allow the compartment fire severity to be represented in a generalised manner across different fire scenarios.

Two key parameters identified in Sect. 2.3 are extracted from the obtained temperature–time curves: (a) the total fire duration, defined as the period during which the temperature exceeds 500 [°C], and (b) the average peak fire temperature. A flashover temperature of 500 [°C] was adopted in this study based on the widely accepted criterion proposed by McCaffrey et al. [35] and the more recent work of Xia et al. [36], both of which identify an upper-layer gas temperature of approximately 500 [°C] as indicative of flashover. This assumption is consistent with established literature, while adopting a lower flashover temperature would result in a more conservative assessment.

Histograms of total fire duration and the average peak fire temperature are shown in Fig. 4. The results indicate that most compartment fires have total flashover durations of less than 100 min and peak temperatures below 1200 [°C].

### Data Preprocessing for Training Purposes

As the full-scale compartment fire tests were originally conducted for a range of different research objectives, not all available data are suitable for the current study. The primary objective of this work is to determine which fire model (the EN parametric fire model or the TF model) provides a better representation of natural compartment fires under specific conditions. Therefore, the data selection process needed to satisfy the assumptions and applicability limits of both fire models, as well as the requirements of the LR model.

The following selection criteria were applied to the experimental data. The compartment floor area had to exceed 10 [m$^2$], representative of typical office or residential compartments. The fire load density and ventilation factor were required to be greater than 50 [MJ/m$^2$] and 0.01 [m$^{0.5}$], respectively, corresponding to the lower applicability limits of the EN parametric fire model [39]. In addition, the peak fire temperature had to exceed 500 [°C] so that the total fire duration could be determined, and the cooling phase had to be at least partially recorded for the same reason. The measurements from repeated tests are averaged.

**Fig. 5:**
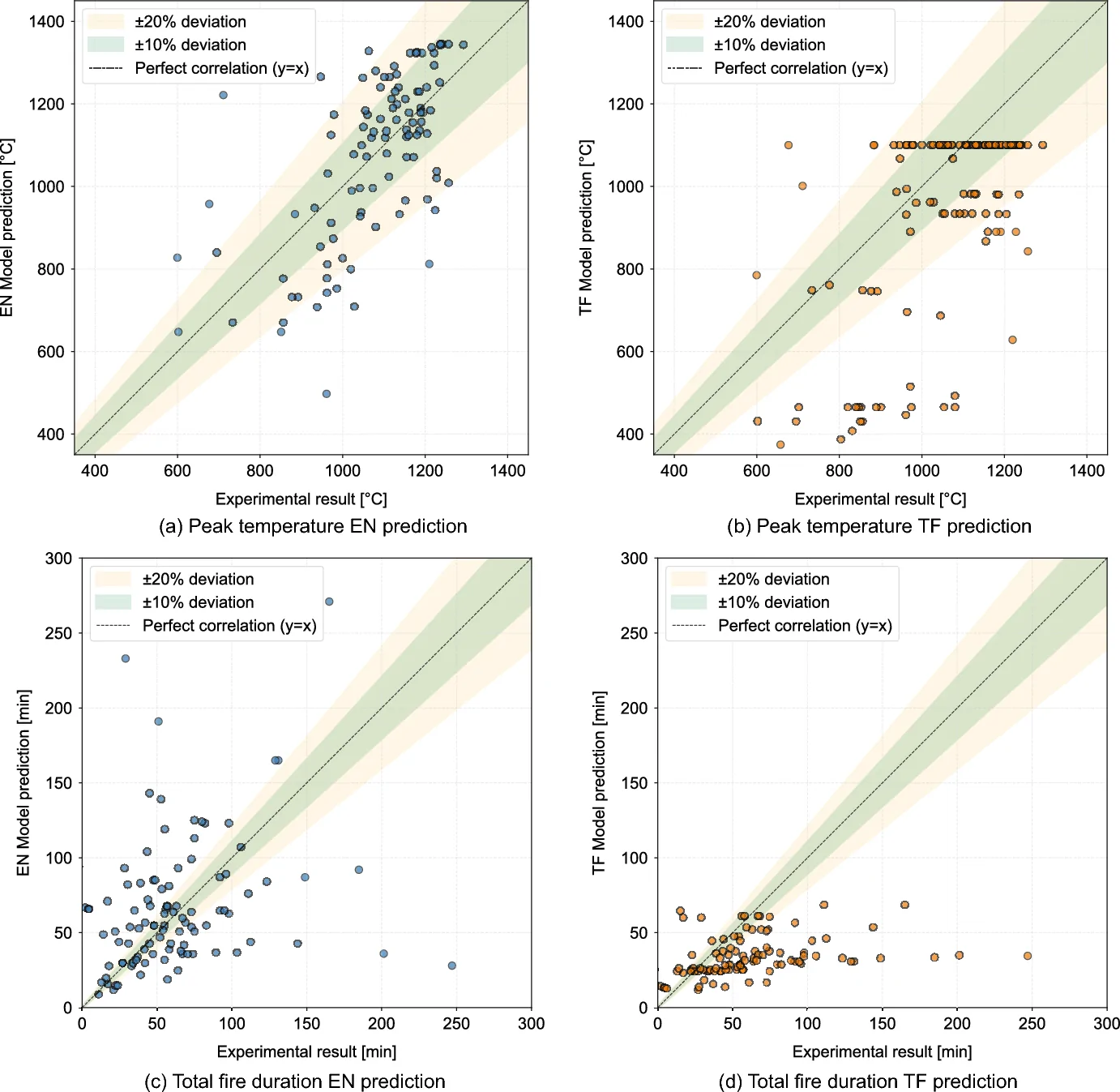
Comparison of EN and TF model predictions with experimental peak temperature and total fire duration

Based on the selection criteria described above, 138 out of 222 full-scale natural fire experiments are selected to train the LR model. Following the third step of the proposed methodology described in Sect. 2.1, each selected fire experiment is simulated using both the EN parametric fire curve and the TF model to evaluate their predictive performance. Fig. 5 presents an example comparison between the model predictions and the experimental results. For this example, a constant heat release rate per unit area of 250 [kW/m$^2$] (recommended for office buildings in EN 1991-1-2 Table E.5 [16]), a constant fire spread rate of 2 [mm/s] [9, 40], and a constant peak flame temperature of 1100 [°C] [41, 42] are adopted for the TF model. Note that to develop an LR model that is applicable to a wider range of fire scenarios, these TF model parameters are subsequently treated as variables rather than fixed values. The heat release rate per unit area is varied from 150–650 [kW/m$^2$] [43], the peak flame temperature from 800–1200 [°C] [25], and the fire spread rate from 1.5 to 20 [mm/s] [25]. As demonstrated later in Sect. 4.2, this enables the proposed LR model to remain robust across a wider range of fire scenarios.

In Fig. 5, data points located above the perfect correlation line indicate over-prediction by the model, while those below indicate under-prediction. In general, the EN model over- and under-predicts peak fire temperatures and total fire duration, and most data points fall within ± 20% of the perfect-correlation line for peak-temperature prediction. The TF model tends to under-predict these fire experiments in general, but this does not imply that it performs worse than the EN model. There are two main reasons: (a) because a larger number of tests here have floor areas smaller than 100 m$^2$ (see Fig. 4), which generally favours the EN model and can bias the visual comparison. This imbalance arises because full-scale tests with large floor areas are expensive, leading to fewer such experiments. (b) The TF model is applied here as published in [17], retaining its standard simplifying assumptions of a fixed peak temperature and a constant fire spread rate. As these input parameters are difficult to determine during the design stage but might influence the LR model determination, the next section shows a sensitivity study to evaluate whether the resulting assumptions affect the robustness of the proposed LR model.

## Logistic Model Training and Performance

### Training Procedure

The model training procedure consists of three steps. First, the best-performing fire model is determined for each test. Second, the LR classifier is trained using the results from the first step. Third, these two steps are repeated as part of a sensitivity study to identify the most robust LR model.

In Step 1, the predicted peak fire temperatures and total fire durations from the EN and the TF model are compared against full-scale natural fire experiments by calculating their relative errors, defined as:

24 $$\begin{aligned} \text {Relative\,Error} = (\text {Prediction} - \text {Test}) / \text {Test} \end{aligned}$$

Subsequently, the total relative error for the EN and TF models is calculated as the weighted sum of the relative errors in peak fire temperature and total fire duration ($W_T \times$ peak temperature error $\ +\ W_d \times$ total fire duration error), where $W_T = 0.368$ and $W_d=0.252$ are the weights for peak temperature and fire duration, respectively. These weights are taken from the sensitivity study from Sect. 2.3. The model with the lower absolute total relative error is considered to be the best-performing fire model. Fire experiments in which both models fail to produce valid results are excluded from further steps. The resulting labelled dataset serves as the training data for the LR model.

In Step 2, a LR model is trained to predict the best-performing fire model based on five input parameters: fire load density, floor area, thermal inertia, opening factor, and compartment height. All input features are standardised before training. The resulting LR coefficients, *β *, are then determined and used in Eq. 21.

**Fig. 6:**
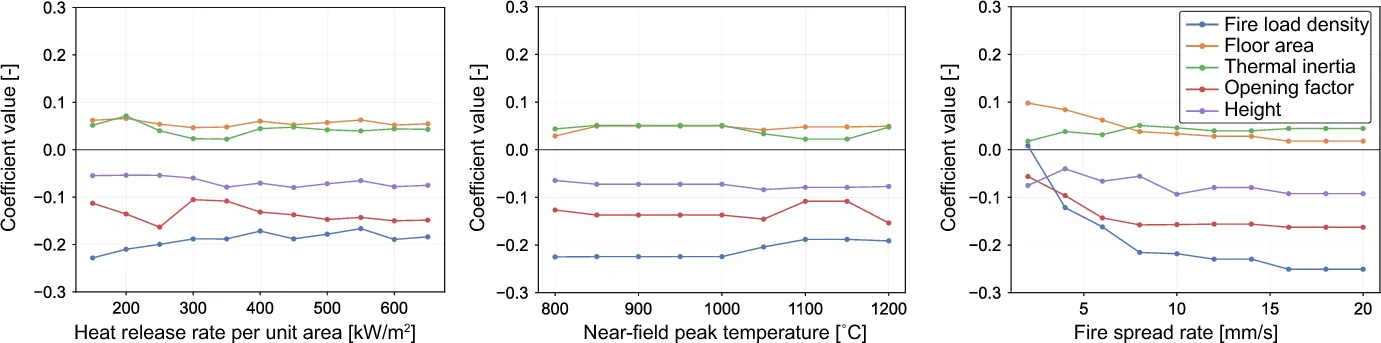
Sensitivity of the LR coefficients to the TF model assumptions

In Step 3, as fixed values are adopted for the heat release rate per unit area, peak flame temperature, and fire spread rate in the TF model used in Step 2, these parameters could influence the selection of the best-performing model and, in turn, the resulting coefficients *β * in LR model (Eq. 21). Therefore, a sensitivity study is conducted to evaluate the influence of different combinations of these input parameters on the coefficients of the LR model. The following TF input ranges are considered: heat release rate per unit area from 150 to 650 [kW/m]$^2$, peak flame temperature from 800 to 1200 [°C], and fire spread rate from 1.5 to 20 [mm/s]. The effects of these assumptions to the resulting coefficients are shown in Fig. 6.

The results show that the selection of the TF input parameters has a substantial influence on the final LR model. The effects of the heat release rate and peak fire temperature are relatively small, whereas the assumed fire spread rate has a significant influence on the resulting LR coefficients (Fig. 6 on the right). When a low fire spread rate is assumed, the LR model identifies floor area as the dominant parameter distinguishing between the EN and TF models. This behaviour is reasonable because, according to the assumptions of the TF model, a low fire spread rate represents a small, localised fire. Consequently, for a small floor area, the burning area is also small, resulting in a very low predicted peak fire temperature. However, when a high fire spread rate is assumed, the TF model behaves more like a whole-compartment fire, and fire load density and opening factor become the dominant distinguishing parameters. Under these conditions, the primary difference between the EN and TF models is governed by the fire load density and ventilation conditions.

Therefore, it is difficult to develop a single LR model without accounting for the assumed fire spread rate. As this parameter is generally difficult to determine in practice, three LR segments are proposed for distinguishing between the EN and TF models, each corresponding to a different assumed fire spread rate in the TF model, as shown in Eq. 25.

25 $$\begin{aligned} z = {\left\{ \begin{array}{ll} -0.003 + \dfrac{598.27-q}{5060.7} + \dfrac{A_f-74.67}{720.8} + \dfrac{b-806.32}{7167.8} & \\ \quad + \dfrac{0.060-O}{0.344} + \dfrac{2.87-H_c}{12.26}, & S < 4.0 \ \text {mm/s} \\ -0.005 + \dfrac{572.76-q}{1624.1} + \dfrac{A_f-72.05}{1348.3} + \dfrac{b-812.18}{10075.1} & \\ \quad + \dfrac{0.059-O}{0.268} + \dfrac{2.86-H_c}{9.30}, & 4.0 \le S \le 6.0 \ \text {mm/s} \\ -0.008 + \dfrac{552.55-q}{1327.9} + \dfrac{A_f-69.87}{1840.5} + \dfrac{b-815.71}{8055.2} & \\ \quad + \dfrac{0.058-O}{0.240} + \dfrac{2.84-H_c}{15.57}, & S \ge 6.0 \ \text {mm/s} \end{array}\right. } \end{aligned}$$

where *q* = fire load density [MJ/m²], $A_f$ = floor area [m²], *b* = thermal inertia [J/(m²·s$^{0.5}$·K)], *O* = opening factor [m$^{0.5}$], $H_c$ = building height [m], and *S* is the assumed fire spread rate. Once the linear predictor *z* is obtained, the probability *p* that the TF model to be the best-performing model for the given scenario can be calculated using Eq. 22.

### Model Performance

**Fig. 7:**
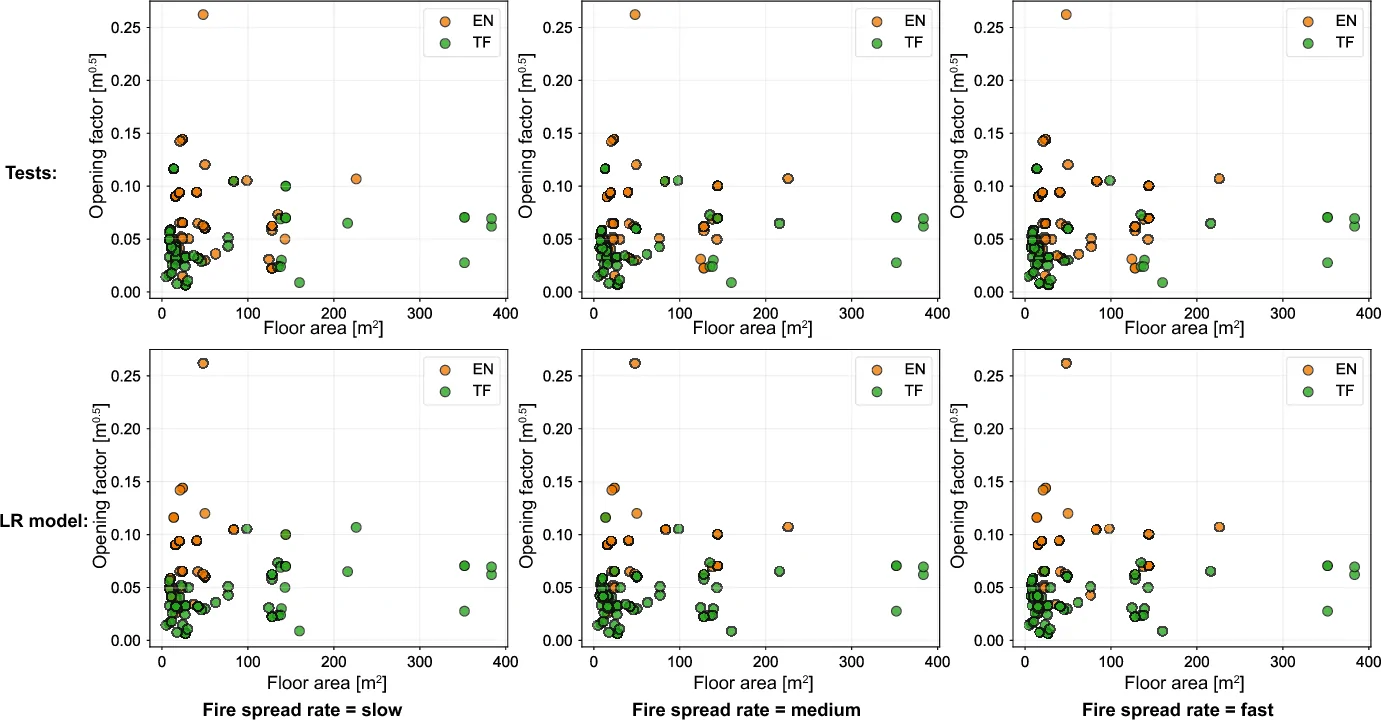
Performance of the trained LR model

The proposed LR model sacrifices some prediction accuracy in order to improve its robustness across a wide range of fire scenarios, as discussed in Sect. 4.1. Three fire spread rates are considered in this study. The predicted best-performing fire model (EN or TF) for the experimental tests is shown in the first row of Fig. 7, while the corresponding LR model predictions are shown in the second row. The corresponding prediction accuracies, coefficients of variation (COVs), and ROC–AUC values are summarised in Table 2. The reported prediction accuracies are based on the deduplicated dataset; without removing duplicated cases, the prediction accuracy would be overstated by approximately 3–5 percentage points.

**Table 2:** Accuracy and coefficient of variation (COV) for the three spread-rate-specific reference LR models

Spread rate range [mm/s]	Training accuracy [%]	COV [%]	ROC-AUC [%]
Slow: *S ≤ 4.0*	71.6	3.21	75.8
Medium: *4.0 < S ≤ 6.0*	74.2	1.35	81.2
Fast: *S > 6.0*	75.0	2.21	85.6
Average	73.6	3.07	80.8

The first row of Fig. 7 shows that the predicted best-performing fire model is relatively insensitive to the assumed fire spread rate. Most changes occur for floor areas between 100 and 150 [m$^2$], indicating that the predicted differences between the EN and TF models are small in this range and therefore difficult to distinguish. In other words, either fire model provides a reasonable prediction. For floor areas greater than 150 [m$^2$], the TF model consistently becomes the best-performing model, and the assumed fire spread rate has small influence on the prediction. This is consistent with the underlying physical assumptions of the TF model, which represents fire development as a localised burning process.

In terms of prediction accuracy (the second row of Fig. 7; Table 2), the LR model shows the lowest accuracy for the slow fire spread rate. This is because for a fixed local burnout time, the actively burning length in the TF model scales with the assumed fire spread rate. A slower spread rate therefore produces a smaller burning area, lower heat release, and lower fire temperatures, which more frequently fail to reach the threshold required for comparison, resulting in a larger proportion of "No Winner" cases for predicting the best-performing model. This reduces the separability between the EN and TF models rather than reflecting a limitation of the LR model. As the fire spread rate increases, the TF model behaves more like a whole-compartment fire, producing a more stable and physically distinguishable comparison with the EN model. Consequently, the LR model receives a clearer classification signal and achieves higher prediction accuracy.

## Implementation of the Model in Fire Engineering

In this section, the practical implementation of the proposed LR model in performance-based fire engineering design is demonstrated. By combining the model with Monte Carlo simulation, the fire risk of the design can be quantitatively evaluated via the failure probability, which in turn enables the calculation of structural reliability and risk-based fire performance periods. Similar risk-informed design frameworks have been reported in the literature [34, 44, 45].

It should be noted that this section aims to demonstrate the application and performance of the developed LR model, rather than to provide recommendations for fire-safe design decisions. Therefore, the illustrative example presented here does not strictly comply with current design guidelines. A comprehensive design-oriented application is beyond the scope of this study and is left for future work.

### Implementation of the LR Model

Appendix A briefly demonstrates the use of the trained LR model in Python, which can be readily integrated into the risk-based approach introduced later in this section. For the reader’s reference, an example script implementing the risk-based approach is provided in Appendix B, and the trained model file, $fire\_model\_selector.pkl$, is available in Appendix C.

The model is first loaded from the pickle file, which contains the trained classifier, feature names, and associated data scaler. For a given fire scenario, the five aforementioned input parameters should be provided. The trained model then automatically normalises the inputs, evaluates the scaled values, and computes the probability for each fire model using Eq. 25. The outputs are the probabilities *p* of the EN and TF models, respectively. Scenarios with $P(\textrm{TF}) \ge 0.5$ are assigned to the TF model, while scenarios with $P(\textrm{TF}) < 0.5$ are assigned to the EN fire model.

**Fig. 8:**
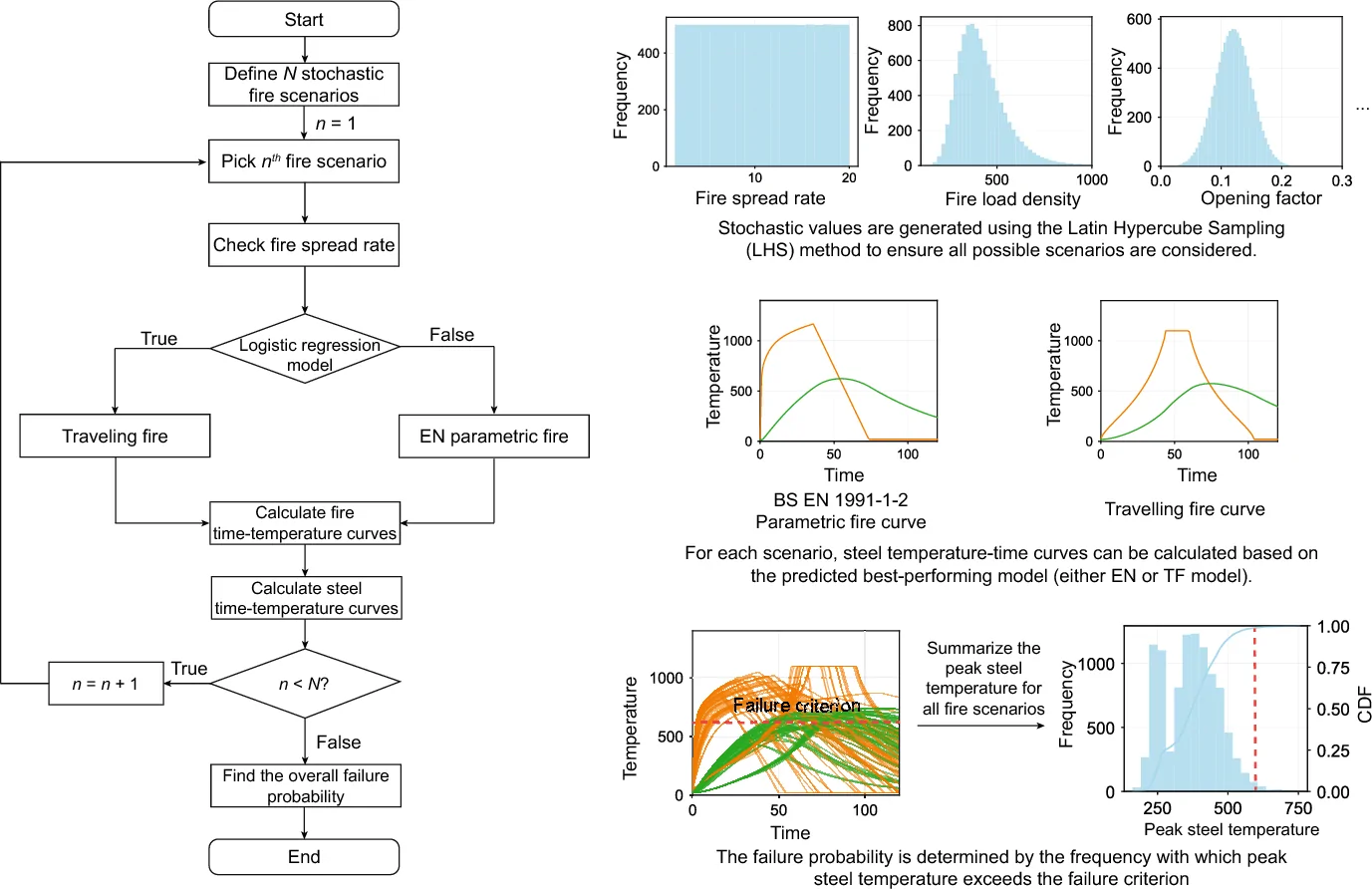
Flowchart of implementing the LR model in fire design

The flowchart for implementing the developed LR model in risk-based fire design is shown in Fig. 8, with the Monte Carlo simulation described in Sect. 2.5.

At the beginning of the risk analysis, the total number of simulations, *N*, should be predefined. The value of *N* must be sufficiently large to ensure stable and reliable results. For the six input parameters: assumed fire spread rate, fire load density, floor area, thermal inertia, opening factor, and compartment height, *N* sets of input values are generated according to their prescribed probability distributions. Each set represents one possible fire scenario, resulting in a total of *N* possible fire scenarios (Fig. 8 first row on the right).

For each generated scenario, the given fire spread rate is first evaluated. If it is less than 4.0 [mm/s], the LR model corresponding to the slow fire spread rate is used. If it is between 4.0 and 6.0 [mm/s], the LR model corresponding to the medium fire spread rate is applied. Otherwise, the LR model corresponding to the fast fire spread rate is used. The three LR models are given in Eq. 25. The selected LR model then determines whether the EN or TF model is the best-performing model. The corresponding fire temperature–time curve and steel temperature–time response are subsequently obtained for each scenario (second row of Fig. 8).

This procedure is repeated for all *N* scenarios, and the corresponding peak steel temperatures are obtained and presented as a histogram and Cumulative Distribution Function (CDF) in the last row of Fig. 8. If the calculated peak steel temperature exceeds the failure criterion, the scenario is counted as a failure $N_f$. The overall failure probability is then calculated using Eq. 23 for decision-making.

### Prediction of Structural Failure Probability

#### Model Setup

Following the procedure described in the previous section, an office building is used to demonstrate the proposed framework. The fire spread rate is assumed to be uniformly distributed from 1.5 to 20 [mm/s] by [25]. The fire load density is assumed to follow a Gumbel distribution with a mean value of 420 [MJ/m$^2$] and a COV of 0.3, as recommended by the British guideline PD 7974-7 [46]. Following the approaches of Kirby et al. [44] and Law et al. [45], the compartment floor area is treated as uniformly distributed between 20 [m$^2$] and 200 [m$^2$]. Alternatively, the floor area may be specified as a fixed value when evaluating the failure probability of an individual compartment, as suggested by Lucherini et al.  [47]. Both approaches are applicable, depending on the intended scope of the probabilistic analysis.

The opening factor of a fire compartment depends on the building layout and the breakage of glazing elements during a fire [48]. When considering multiple compartments simultaneously, it is therefore difficult to predict the opening factor simultaneously. After reviewing reported opening factors for different occupancies, the opening factor is assumed to follow a uniform distribution between 0.02 and −0.13 [m$^{1/2}$] [44, 49], representing a wide range of possible scenarios. For practical applications to a specific building, however, the opening factor should be determined based on the building geometry and the expected glazing behaviour during a fire. The thermal inertia is modelled using a normal distribution with a mean value of 423.5 [J/m$^2\cdot$s$^{1/2}\cdot$K] and a COV of 0.09, as recommended by Shi et al. [50] for office buildings. The compartment height is assumed to be 3.5 [m] throughout.

**Fig. 9:**
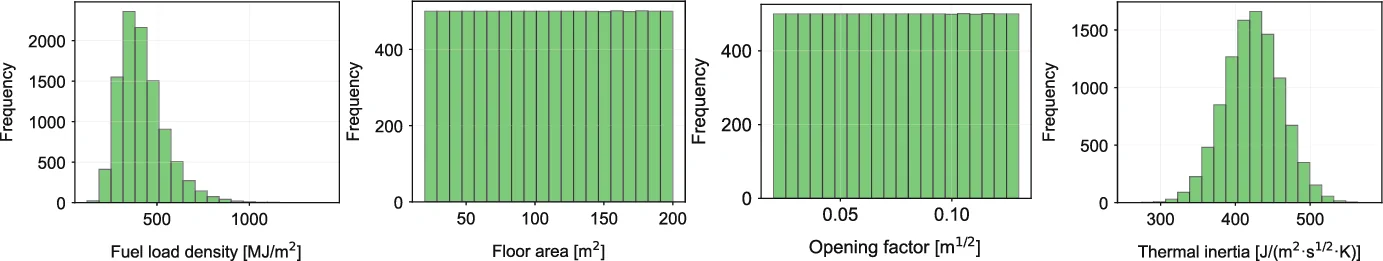
Stochastic inputs for the fire models

A total of 10,000 Monte Carlo simulations is conducted using the aforementioned input parameters. The material properties of the fire-protected steel members are taken to be the same as those used in Sect. 2.3. All samples are generated using Latin Hypercube Sampling to ensure adequate representation of the tails of the distribution. The resulting input distributions are shown in Fig. 9.

#### Results and Discussion

**Fig. 10:**
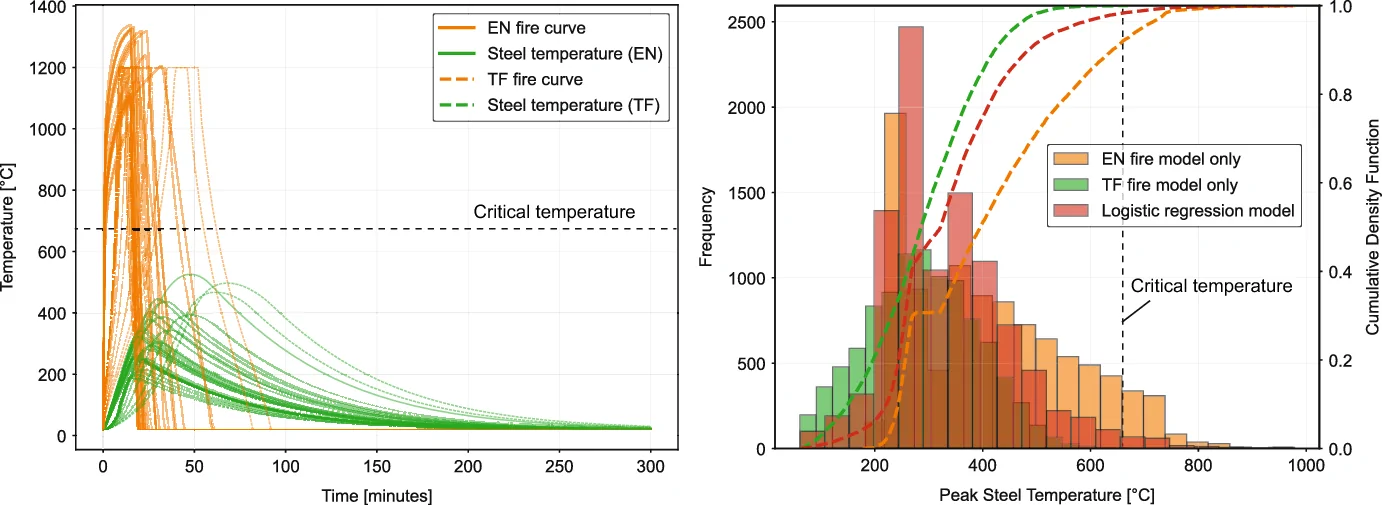
Results of the fire curves and peak steel temperatures

The results from the Monte Carlo simulations (MCs) are shown in Fig. 10. The figure on the left is the randomly selected fire and steel temperature curves using the developed LR model. For this office building, the generated fire scenarios are classified 60% of EN and 40% of TF scenarios. The TF scenarios have a longer fire-growth stage, so the steel peak temperatures occur later than in the EN scenarios.

The figure on the right compares the peak steel temperatures obtained using three modelling strategies: (a) 10,000 times MCs using the EN model only, (b) 10,000 times MCs using the TF model only, and (c) 10,000 times MCs using the LR model combining the EN and TF models. The cumulative density functions show that, compared with the TF model, the EN model generally predicts higher steel temperatures. This suggests that the TF model tends to predict fewer low-severity fire scenarios and, consequently, lower failure probabilities when relatively high failure criteria are used. As the structural load is not considered here, the temperature failure criterion of 650 [°C] [51] is used. The calculated failure probabilities are 9.15% for the EN fire model, 0.02% for the TF fire model, and 1.93% for the LR model.

Full-scale fire test results (Fig. 7) already show that using only the TF or EN fire model does not always accurately capture the compartment temperature, which can lead to an overestimation of fire risk for some fire scenarios. When lower failure criteria are used, the differences in predicted failure rates between the EN-only and TF-only models become more significant. Therefore, as shown in Fig. 10, by automatically selecting the best-performing fire model for each scenario, the proposed LR model reduces both over- and under-design, leading to more balanced and reliable risk-based fire designs.

## Conclusions and Future Work

This study summarises 222 available full-scale compartment natural fire experiments. By comparing the experimental setups with the prediction performance of two fire models, an LR model is trained to statistically identify the threshold between the EN parametric fire curves and the travelling fire curves, thereby supporting more informed decision-making in performance-based structural fire design.

When evaluating natural fire curves from the structural fire engineering perspective, a sensitivity study quantitatively shows that the peak fire temperature (36.8%) and the total fire duration (25.2%) are the most influential factors affecting the resulting peak steel temperature, whereas the fire growth phase has a relatively minor influence (less than 2%).

The assumed fire spread rate was found to substantially influence the LR coefficients. Therefore, a framework comprising three LR models, each corresponding to a different assumed fire spread rate, was proposed, achieving an average training accuracy of 0.736 and an ROC–AUC of 0.808. Specifically, when a low fire spread rate is assumed, floor area is the dominant parameter distinguishing between the EN and TF models, whereas fire load density and opening factor become dominant at higher fire spread rates. Based on this analysis, a statistically derived threshold (Eq. 25) is proposed to distinguish between the EN and TF models across different fire scenarios.

Finally, the example application demonstrates that, compared with EN-only and TF-only approaches, the proposed LR model can reduce both over-design and under-design by automatically selecting the best-performing fire model. This leads to a more balanced and reliable risk-based structural fire design.

Regarding future work, the TF model requires further development. At present, key input parameters, such as the peak flame temperature and fire spread rate, must be specified by engineers rather than being predicted from the fire characteristics, as in the EN parametric fire model. Consequently, several assumptions are required when developing the proposed LR model. Improving the physical prediction of these TF model parameters would reduce these assumptions and provide a more physically representative basis for the LR model. In addition, the opening factor distribution adopted in this study is assumed. The proposed risk-based framework could therefore be further improved by incorporating building-specific or statistically derived opening factor distributions when such data become available.

## Figures and Tables

**Algorithm 1:**
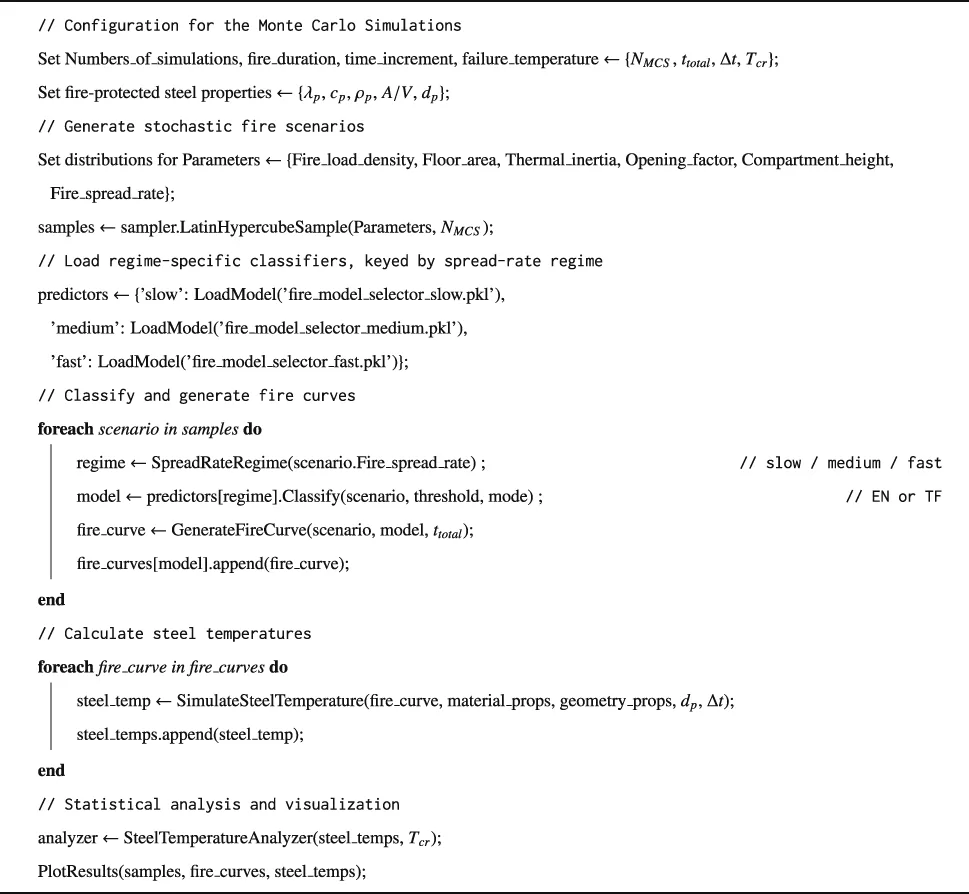
Stochastic Fire Analysis and Steel Temperature Calculation

## Data Availability

No datasets were generated or analysed during the current study.

## Appendix C: Trained LR Model

The trained LR model can be found on GitHub at the following address: *https://github.com/QingfengAF/Logistic_regression_model_EN_TF.git.*

## References

[1] Van Coile R, Hopkin D, Lange D, Jomaas G, Bisby L (2019) The need for hierarchies of acceptance criteria for probabilistic risk assessments in fire engineering. Fire Technol 55(4):1111–1146. 10.1007/s10694-018-0746-7

[2] ISO 834 (1999) Fire-resistance tests—elements of building construction, Standard, International Organization for Standardization, Geneva, Switzerland

[3] Law A, Bisby L (2020) The rise and rise of fire resistance. Fire Saf J 116:103188. 10.1016/j.firesaf.2020.103188

[4] Lucherini A, Jovanovi B, Torero JL, Van Coile R, Merci B (2024) Thermal characterisation of the cooling phase of post-flashover compartment fires. Int J Therm Sci 199:108933. 10.1016/j.ijthermalsci.2024.108933

[5] Quintiere JG (2006) Fundamentals of fire phenomena. John Wiley & Sons, Hoboken

[6] Put F, Lucherini A, Merci B, Van Coile R (2024) Model uncertainty in a parametric fire curve approach: a stochastic correction factor for the compartment fire load density. Fire Saf J 144:104113. 10.1016/j.firesaf.2024.104113

[7] Cajot L-G, Schleich J-B, Pierre M (1998) Natural fire safety concept: full-scale tests, implementation in the Eurocodes and development of a user-friendly design tool: final report, Technical report, European Coal and Steel Community (ECSC), Luxembourg

[8] Rein G, Zhang X, Williams P, Hume B, Heise A, Jowsey A, Lane B, Torero J (2007) Multi-storey fire analysis for high-rise buildings, in: Proceedings of the 11th International Interflam Conference, Interflam, London, UK, pp 605–616

[9] Kirby B, Wainman D, Tomlinson L, Kay T, Peacock B (1999) Natural fires in large scale compartments. Collaborative project report. British Steel Technical and Fire Research Station, Rotherham, UK

[10] Thomas I, Bennetts I (2000) Fires in enclosures with single ventilation openings—comparison of long and wide enclosures. Fire Safety Science—Proceedings of the Sixth International Symposium, International Association for Fire Safety Science, pp 941–952

[11] Rein G, Abecassis Empis C, Carvel R (2007) The Dalmarnock fire tests: experiments and modelling, technical report, School of Engineering and Electronics, University of Edinburgh, Edinburgh, UK

[12] Welch S, Jowsey A, Deeny S, Morgan R, Torero J (2007) BRE large compartment fire tests—characterising post-flashover fires for model validation. Fire Saf J 42(8):548–567. 10.1016/j.firesaf.2007.04.002

[13] Xu Q, Hofmeyer H, Maljaars J, van Herpen R (2024) Full-scale fire resistance testing and two-scale simulations of sandwich panels with connections. Fire Technol 60:2461–2488. 10.1007/s10694-023-01463-y

[14] Lennon T (2016) BRE Cardington steel framed building fire tests: data release, technical report. Building Research Establishment (BRE), Watford, UK

[15] Xu Q, Hofmeyer H, Maljaars J, van Herpen R (2024) Pyrolysis modelling of insulation material in coupled fire-structure simulations. J Build Eng 98:110969. 10.1016/j.jobe.2024.110969

[16] EN 1991-1-2 (2002) Eurocode 1: actions on structures—part 1-2: general actions—actions on structures exposed to fire, Standard, European Committee for Standardization (CEN), Brussels

[17] Stern-Gottfried J, Rein G (2012) Travelling fires for structural design-part II: design methodology. Fire Saf J 54:96–112. 10.1016/j.firesaf.2012.06.011

[18] Dai X, Welch S, Usmani A (2017) A critical review of “travelling fire’’ scenarios for performance-based structural engineering. Fire Saf J 91:568–578. 10.1016/j.firesaf.2017.04.001

[19] Franssen J-M (2000) Improvement of the parametric fire of Eurocode 1 based on experimental tests results. 6th international Symposium on Fire Safety Science, Curtat ed., Poitiers, France

[20] Cox DR (1958) The regression analysis of binary sequences. J Roy Stat Soc 20(2):215–232. 10.1111/j.2517-6161.1958.tb00292.x

[21] Jafari Goldarag Y, Mohammadzadeh A, Ardakani A (2016) Fire risk assessment using neural network and logistic regression. J Indian Soc Remote Sens 44(6):885–894. 10.1007/s12524-016-0557-6

[22] Eslami R, Azarnoush M, Kialashki A, Kazemzadeh F (2021) GIS-based forest fire susceptibility assessment by random forest, artificial neural network and logistic regression methods. J Trop For Sci 33(2):173–184

[23] Kale ÖA, Baradan S (2020) Identifying factors that contribute to severity of construction injuries using logistic regression model. Teknik Dergi 31(2):9919–9940

[24] Song X (2020) Parameterized fragility analysis of steel frame structure subjected to blast loads using Bayesian logistic regression method. Struct Saf 87:102000. 10.1016/j.strusafe.2020.102000

[25] Rackauskaite E, Hamel C, Law A, Rein G (2015) Improved formulation of travelling fires and application to concrete and steel structures. Structures 3:250–260. 10.1016/j.istruc.2015.06.001

[26] Hopkin DJ (2013) Testing the single zone structural fire design hypothesis. Interflam 2013, proceedings of the 13th international conference. pp 139–150

[27] EN 1993-1-2 (2005) Eurocode 3: design of steel structures—part 1-2: general rules—structural fire design, Technical report, European Committee for Standardization (CEN), Brussels, Belgium

[28] LDV (2005) Pilot project, handbook 5: design of buildings for the fire situation

[29] Moradi MJ, Hajiloo H (2025) Meta-analysis of compartment fires: exploring extensive experimental datasets with heat release rate in focus. Appl Therm Eng 266:125733

[30] Moradi MJ, Hajiloo H (2026) Modeling compartment fire HRR and temperature development: an alternative to the Eurocode parametric fire. Appl Therm Eng. 10.1016/j.applthermaleng.2026.129705

[31] Alpert RL (1972) Calculation of response time of ceiling-mounted fire detectors. Fire Technol 8(3):181–195. 10.1007/BF02590543

[32] Rackauskaite E, Hamel C, Law A, Rein G (2015) Improved formulation of travelling fires and application to concrete and steel structures. Structures 3:250–260. 10.1016/j.istruc.2015.06.001

[33] Fu I, Rickard I, Hopkin D, Spearpoint M (2019) Application of python programming language in structural fire engineering—Monte Carlo simulation. Proceedings of the Interflam 2019 Conference, Royal Holloway College, Egham, UK, pp 1–3

[34] Xu Q, Hopkin C, Wang Y (2026) Uncertainties in natural fire models: accuracy and probabilistic application in structural fire design. Fire Saf J 163:104823. 10.1016/j.firesaf.2026.104823

[35] McCaffrey BJ, Quintiere JG, Harkleroad MF (1981) Estimating room temperatures and the likelihood of flashover using fire test data correlation. Fire Technol 17(2):98–119. 10.1007/BF02479583

[36] Xia T, Ruan H, Wang Y (2024) Predicting the flashover occurrence and energy distribution in compartment fires with different boundary materials. Fire Saf J 146:104183

[37] Moradi MJ, Hajiloo H (2025) Quantitative review of experimental tests and theoretical models of flashover occurrence in compartment fires. Fire Saf J 155:104432. 10.1016/j.firesaf.2025.104432

[38] Alam N, Nadjai A, Charlier M, Vassart O, Welch S, Sjöström J, Dai X (2022) Large scale travelling fire tests with open ventilation conditions and their effect on the surrounding steel structure-the second fire test. J Constr Steel Res 188:107032. 10.1016/j.jcsr.2021.107032

[39] British Standards Institution (2007) PD 6688-1-2:2007 background paper to the UK National Annex to BS EN 1991-1-2. Technical report. British Standards Institution, London

[40] Thomas PH (1967) Some aspects of the growth and spread of fire in the open. Forestry 40(2):139–164. 10.1093/forestry/40.2.139

[41] Audouin L, Kolb G, Torero J, Most J (1995) Average centreline temperatures of a buoyant pool fire obtained by image processing of video recordings. Fire Saf J 24(2):167–187. 10.1016/0379-7112(95)00021-K

[42] Drysdale D (2011) An introduction to fire dynamics. John wiley & sons, Hoboken

[43] British Standards Institution (2019) PD 7974-1:2019 application of fire safety engineering principles to the design of buildings. Initiation and development of fire within the enclosure of origin (sub-system 1), Technical report. British Standards Institution, London

[44] Kirby B, Newman G, Butterworth N, Pagan J, English C (2004) A new approach to specifying fire resistance periods. Struct Eng 82(19):1

[45] Law A, Stern-Gottfried J, Butterworth N (2015) A risk based framework for time equivalence and fire resistance. Fire Technol 51(4):771–784. 10.1007/s10694-014-0410-9

[46] PD 7974-7:2019 (2019) Application of fire safety engineering principles to the design of buildings—part 7: probabilistic risk assessment. British Standard

[47] Lucherini A, Ni S, Unobe D, Gernay T, Chaudhary RK, Peng M, Van Coile R (2024) Application of cost-benefit analysis methodology for fire safety measures in structural fire engineering. 13th International Conference on Structures in Fire (SiF 2024), CMM-Portuguese Steelwork Association, pp 25–36

[48] Joint Committee on Structural Safety (JCSS) (2001) Probabilistic Model Code, https://www.jcss-lc.org/jcss-probabilistic-model-code/, part 1: basis of design; part 2: load models; part 3: resistance models

[49] Xie Q, Xiao J, Gardoni P, Hu K (2019) Probabilistic analysis of building fire severity based on fire load density models. Fire Technol 55(4):1349–1375

[50] Shi K, Guo Q, Jeffers A (2013) Stochastic analysis of structures in fire by Monte Carlo simulation. J Struct Fire Eng 4(1):37–46. 10.1260/2040-2317.4.1.37

[51] Inerhunwa I (2021) Reliability based analysis of effects of fire service intervention and sprinklers on building fire resistance. The University of Manchester, Phd thesis

